# The health and quality of life of Thalidomide survivors as they age – Evidence from a UK survey

**DOI:** 10.1371/journal.pone.0210222

**Published:** 2019-01-16

**Authors:** Elizabeth Newbronner, Caroline Glendinning, Karl Atkin, Ruth Wadman

**Affiliations:** 1 Department of Health Sciences, University of York, York, United Kingdom; 2 Social Policy Research Unit, University of York, York, United Kingdom; Centers for Disease Control and Prevention, UNITED STATES

## Abstract

**Background:**

In the late 1950s and early 1960s the drug Thalidomide was marketed across the world as a non-addictive tranquilizer. Despite being given to pregnant women as a safe treatment for morning sickness, Thalidomide caused serious damage to the unborn child. Much has been written about the drug and the birth defects it caused but evidence about the health of Thalidomide survivors as they age is limited.

**Aim:**

The aim of this study was to: explore the health and wellbeing UK Thalidomide survivors; document the health problems experienced by them as they reach their mid-50s; and examine the impacts on their health-related quality of life and employment.

**Methods:**

A health and wellbeing survey of 351 UK Thalidomide survivors, which gathered information about home and employment circumstances, recent health problems, and health related quality of life (using SF12 Health Survey). Overall analysis focused on descriptive statistics; the association between respondents’ health related quality of life and original impairment was examined using Pearson Correlation; and a three step Hierarchical Regression was used to explore the influence of five factors which narrative responses suggested might be important.

**Results:**

As Thalidomide survivors reach their mid-50’s they are experiencing a wide range of secondary health problems, in particular musculoskeletal problems, and depression and anxiety, with multimorbidity a growing issue. These health problems are having a negative impact on their employment (two fifths are unable to work) and their physical health related quality of life, which is significantly poorer than the general population.

**Discussion:**

Having lived relatively independent lives, many Thalidomide survivors are now having to adjust to growing disability. The study provides further evidence of the accumulative impact of disability over peoples’ lifetimes and highlights the value of a life course perspective in understanding the complex experience of growing older with a disability.

## Introduction

In the late 1950s and early 1960s the drug Thalidomide was marketed across the world as a non-addictive tranquilizer. Despite being given to pregnant women as a safe treatment for morning sickness, Thalidomide caused serious damage to the unborn child when taken during the first trimester. Much has been written about the drug and the birth defects it caused but evidence about the health of Thalidomide survivors as they age is limited. The wider literature on the experience of ageing with disability also remains relatively sparse, although there has been growing interest in the past decade, in part because the number of people with early acquired disabilities who are reaching mid and later life is increasing.

The aim of this study was to: explore the health and wellbeing UK Thalidomide survivors; document the health problems experienced by them as they reach their mid-50s; examine the impacts on their health-related quality of life and employment; and, where possible, make comparisons with the incidence of similar problems in the general population, and with research on Thalidomide survivors in other countries. By focusing on an identifiable group who are ageing together, the study was also designed to contribute to the wider understanding of ageing with early onset disability.

Our findings illustrates the challenges of ageing with lifelong impairments, particularly the interactions between original, congenital impairments and subsequent ageing processes. They shows the compounding and accumulated impact of impairment and ageing, in which the biological connects to the social, thereby exposing individuals to environmental factors that further generate the potential for disadvantage and social exclusion [1]. These include access to paid work and self-care, previously characterised by high levels of personal independence. As former adaptive mechanisms are compromised, new experiences of disability arise [2].

### Background

Between 1958 and 1961, Thalidomide was widely prescribed in the UK as a safe, none addictive sedative and tranquiliser. It was manufactured by a German company, Chemie Grünenthal, and distributed in the UK by Distillers Company (Biochemicals) Limited. Despite being marketed as an entirely safe treatment for the discomforts of pregnancy (including morning sickness), Thalidomide caused serious damage to the unborn child when taken during the first trimester. Depending on the timing and level of ingestion, foetal damage typically included:

- Missing, shortened or malformed upper limbs–ranging from complete absence of the arm (upper limb Amelia) with one or more digits attached directly to the shoulder, to short or missing long bones and missing or damaged thumbs and fingers
- Shortened or malformed lower limbs–including short or missing long bones, congenital dislocation of the hip, club foot, and extra toes
- Eye, ear and facial damage–these are the second most common group of birth defects and include missing or damaged ears, narrow ear canals, small or damaged eyes, restricted eye movement and facial palsy
- Malformation of internal organs–such as damage to the heart, urinary and alimentary tracts, and reproductive organs [3].

Together, these birth defects are referred to as Thalidomide Embryopathy or Thalidomide Syndrome.

Thalidomide constituted a major medical safely scandal and has, in Sarah Ferber’s [4] words become *“emblematic of the advance then shocked reversal of the optimism of the ‘pharmaceutical revolution’ of the mid twentieth century”* (p133). In the UK it prompted extended public campaigns by the Sunday Times newspaper and the parents of UK Thalidomide-damaged children for recognition and compensation. It led to global changes in the way in which drugs are tested and regulated [5], and in the UK to: the establishment of a Royal Commission on Civil Liability and Compensation for Personal Injury; changes to definitions of contempt in civil law cases; and a Law Commission report and legislation on civil liability in ante-natal injury.

A charitable trust—the Thalidomide Children’s Trust (now the Thalidomide Trust–see www.thalidomidetrust.org), was established to oversee compensation payments from Distillers to the families affected by Thalidomide in the UK. Individual payments were determined through assessments of level of impairment, with points being given for different types and severity (totalling from 3.5 to 75 points). The Thalidomide Trust continues to distribute annual payments from the global company Diageo (successor to Distillers) to all UK born Thalidomide survivors. An elected beneficiary National Advisory Council (NAC) advises the Trust. Subsequent campaigns by Thalidomide survivors themselves have focused on the UK Government’s ill-judged licensing of the drug, which had not been adequately tested. In response, in 2010 the Departments of Health (DH) in England, Scotland, Wales and Northern Ireland began paying an annual Health Grant to Thalidomide survivors, in recognition of their growing health and health-related needs. The DH grants, which have been agreed until April 2022, are also administered by the Thalidomide Trust and distributed annually in line with the severity of initial impairment.

Compared to Thalidomide survivors worldwide, UK survivors are relatively unusual in that their families, (and now they themselves) have received compensation payments from childhood. The level of this compensation has increased significantly over the past decade, as a result of both increased payments from Diageo and the DH Grants. This should offer a degree of protection against the disabling consequences of impairments [6]. In other countries compensation arrangements have been much poorer. In Canada for example, when an out of court settlement was reached between Richardson Merrell and 26 families, the company imposed a strict secrecy clause, which resulted in wide disparities in compensation amounts for individuals with similar levels of impairment [5]. In Spain Thalidomide survivors have never received any compensation from the company that made the drug. However, in 2011, after a long campaign by Avite (the Spanish Thalidomide group), the Spanish government did make a small payment to 23 Thalidomide survivors; a fraction of the number believed to have been born in Spain [7].

### Ageing with Thalidomide Embryopathy and ageing with disability

The combination of impairments experienced by many Thalidomide survivors (particularly limb difference) is rare, and the historical, social and legal context of their experience is unique. However, because they are such an identifiable group who are ageing together, comparing the similarities and differences between their experiences of ageing with those of other groups with early onset disability may offer new insights into disability and the life-course.

Evidence about the health of Thalidomide survivors as they age is limited [8]. In particular, few studies have explored the interactions between original Thalidomide impairment, newly emerging secondary damage and co-morbidities. There has also been little attempt to understand how changes to impairment impact on the disabling experience. In the 2000s, when Thalidomide survivors reached their 50s, more research into the health of Thalidomide survivors, began to be undertaken. Whilst many studies have a narrow biomedical focus, together they do suggest widespread age-related secondary damage, particularly musculoskeletal problems (often related to overuse of ‘good’ limbs) and common mental health disorders, notably depression and anxiety [9, 10, 11]. These two conditions may of course be related, if emerging musculoskeletal conditions and consequent loss of function (especially difficulties with activities of daily living and/or reduced ability to work) place Thalidomide survivors at greater risk of mental health problems [12].

In the broader field of disability research, the past decade has seen growing interest in the experience of ageing with disability [13, 14], not least because the number of people with from birth or early acquired disabilities who are reaching middle and older age is increasing [15, 16]. Several overarching themes emerge from the current literature. First, people ageing with disability often face particular difficulties such as secondary conditions and overuse syndromes, post onset syndromes (e.g. post-polio syndrome) and a sense of premature ageing. As Molton and Yorkston [16] note, even in conditions which are regarded as ‘static’ (e.g. Cerebral Palsy), peoples’ functional limitations can change across the life course, creating further potential for disability. Moreover, they suggest that *“there is now mounting evidence that the cumulative effects of living with a disability condition for many years contribute to premature declines in health”* (p291). Secondly, people experiencing the later life effects of early acquired disabilities appear to be more at risk of depression and lower life satisfaction [15,17], although there is evidence that poor overall health, rather than the extent of (original) impairments is more significant in terms of social isolation [13]. Lastly, people growing older with disability often have an elevated risk of acquiring age-related chronic conditions [18, 19], which disability may in turn make more difficult to manage.

Within the disability literature, a few authors [1, 14, 20, 21] have proposed that a life course approach might provide a productive and more holistic framework for thinking about how disability affects people at different points in their lives, including ageing with disability. As Naidoo et al. [14] point out, *“age and disability are not defining traits of an individual but overlapping phenomena that occur throughout the span of the life course”* (p3). Drawing on the principles of life course theory developed by Elder et al. [22], they suggest that a life course perspective brings to the fore *“individual choice and circumstance”* and locates them *“within larger social and historical context”* (p3)

This paper contributes to the literature on ageing with disability by focusing on the experience of UK Thalidomide survivors. It presents evidence that the health problems caused by Thalidomide are not restricted to the well-documented neonatal impairments. On the contrary, it argues that: survivors are encountering significant *new* Thalidomide-related challenges to their physical and mental health and well-being as they age; that these changes in health are linked to individual choice and circumstance; but also to the social and historical context in which Thalidomide survivors have lived their lives.

## Methods

The UK Thalidomide Trust, which holds details of all those to whom it makes Diageo and DH payments, commissioned a survey of the health and wellbeing of its 467 beneficiaries to help develop appropriate support for beneficiaries as they aged. The survey was primarily intended to gather information to inform the Trust’s work and specifically aimed to:

- Quantify the incidence of beneficiaries’ health problems
- Examine beneficiaries’ mental and emotional wellbeing
- Investigate beneficiaries’ wider circumstances and anticipated future needs.

Survey questions were developed in collaboration with the Trust’s Research Committee, Trust staff and NAC. They covered nine main topics: family and housing; work and pensions; original Thalidomide impairments; mobility and equipment; health problems; use of health services; social care support; emotional wellbeing; and health related quality of life. This final topic was explored using the Short Form 12 Health Survey (SF12), a 12-item version of the Short Form-36 Health Survey Questionnaire, widely used in health research in the UK and internationally, to measure health related quality of life. This paper draws on the survey data about respondents’ characteristics and then focuses on two topics—health problems and health related quality of life. Data on the other topics explored in the survey have proved valuable to the Thalidomide Trust but are not discussed here.

The self-completion questionnaire was piloted online and on paper with ten beneficiaries with a range of impairment severity. Paper copies of the final version were sent to all beneficiaries in early August 2015 together with an information sheet and covering joint letter from the Trust Director and NAC Health and Wellbeing Committee Chair. Beneficiaries who had previously informed the Trust they were willing to be contacted by email were also sent the link to an online version; the option of a telephone interview with a member of the research team was also offered. One reminder letter was sent and the survey closed end September 2015 with responses from 351 Thalidomide survivors (75% response rate). Four responses were completed by family members/guardians on behalf of beneficiaries. Although respondents could opt to complete the survey anonymously, 87% gave their names, which enabled their survey responses to be linked to information held by the Trust about their impairment level and country of residence.

The overall analysis–using SPSS (version 24) focused on descriptive statistics. We also examined the association between respondents’ health related quality of life and original impairment using Pearson Correlation, and then used a three step Hierarchical Regression to explore the influence of other factors, which the narrative responses in the survey had suggested might be important (i.e. step 1—original impairment level; step 2—being unable to work and qualifications; and step 3—gender and living alone). Multicollinearity checks were run on all predictor variables included in the models. Where possible and appropriate, the findings from the survey were compared with the general population of a similar age and/or to findings from studies of the health of Thalidomide survivors in other countries. Comments and additional information provided in free text boxes were analysed thematically. This are not reported here in detail but quotations from these narrative responses are used to illustrate findings form the survey.

Ethical approval was obtained from the Department of Health Sciences Research Governance Committee, at the University of UK on the 19 May 2014.

## Results

### Survey respondent characteristics

**Fig 1:**
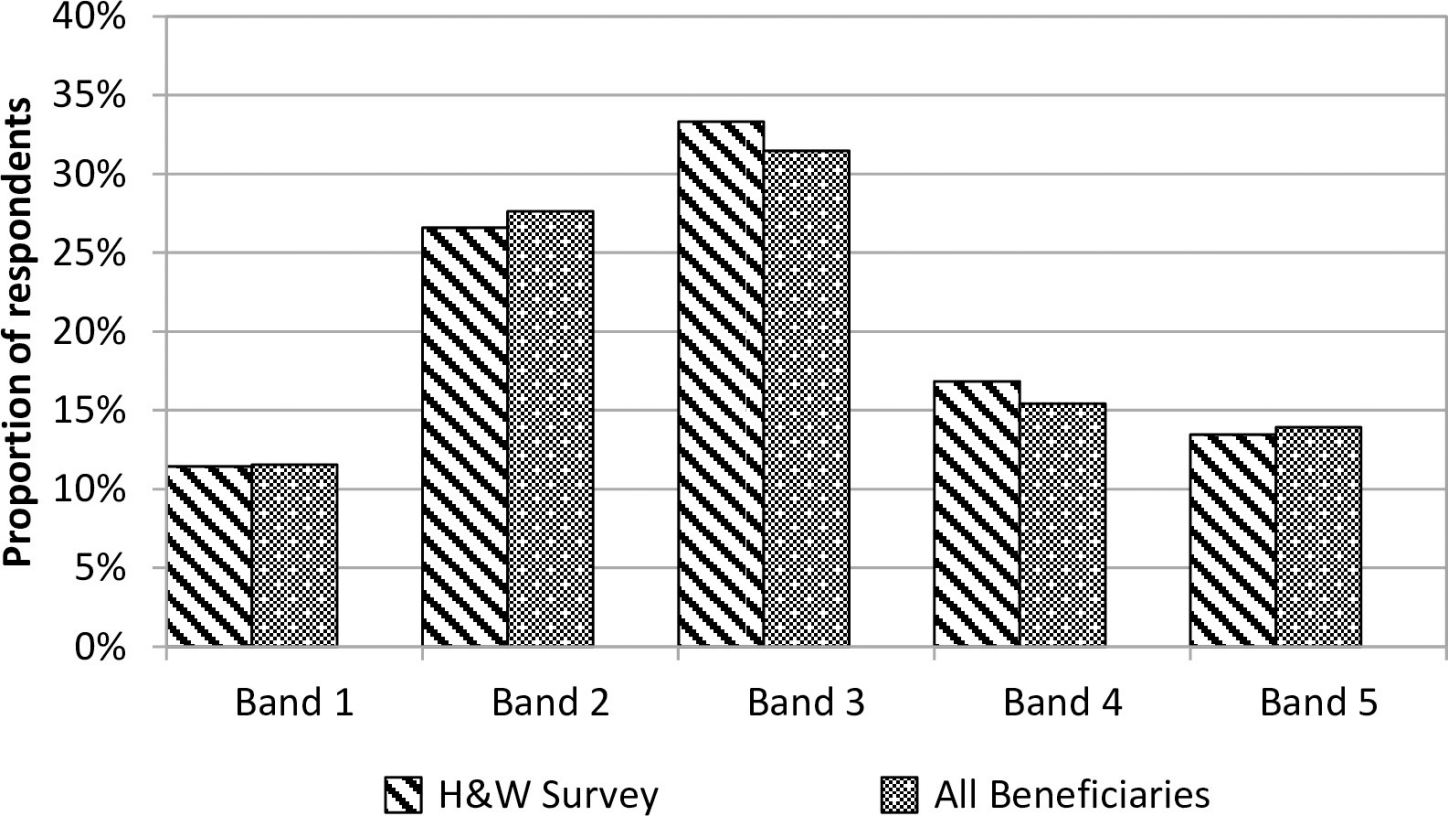
Distributions of survey respondents and all beneficiaries by impairment band. NOTE: n = 302; it was only possible to identify the impairment band for respondents who gave their names.

Here we describe the characteristics of the survey respondents, in order to provide context for the results presented in later sections. In the UK and internationally, limb defects, particularly missing or short arms, are the most commonly recognised feature of Thalidomide damage. However, damage took many forms and varied considerably in severity. The drug was also no respecter of class or culture, so survivors are a widely heterogeneous group. Respondents were evenly split between women and men (174 respectively; three respondent did not give their gender). At the time of the survey, the Trust grouped its beneficiaries in to five impairment bands, with band 1 covering beneficiaries with the least severe impairments and band 5 those with the most severe. Based on these bands, the distribution of the overall severity of their impairments was similar to that of all UK beneficiaries (Fig 1).

Respondents were asked to describe their original Thalidomide impairments using 25 categories covering limb damage, sensory impairments and internal organ damage, plus a free text box for other damage. For ease of analysis, these were collapsed into eight groupings and their incidence compared (in aggregate form) with data held by the Trust. This enabled us to check that the survey respondents were broadly representative, in terms of the range of impairment, of all UK Thalidomide survivors. For example, the proportions in survey and in the Trust’s data were very similar for respondents with upper limb only damage (survey 58%/Trust 54%), lower limb only damage (survey 5%/Trust 4%), and no limb damage, (survey 12%/Trust 11%). However, there was a difference in the proportion with both upper and lower limb damage (survey 25%/Trust 34%).

However, because the Trust’s information was collected at the time beneficiaries were assessed for their compensation claims i.e. from the 1970’s onwards, the survey also provided a more up to date picture of impairments, particularly damage to internal organs which for many had only come to light later in life as a result of diagnostic scans. This was reflected in survey results. For example, 31% of survey respondents said they had damage to internal organs, whereas the Trust’s data showed just 22% with this type of damage. Thalidomide survivors who were blind or partially sighted appear to be under represented in the survey. This may reflect the method of data collection, although large print version of the questionnaire were available and respondent could choose to complete the survey by telephone. Importantly, the survey confirmed that many Thalidomide survivors have multiple impairments e.g. 73 respondents had upper limb damage and were deaf/partially deaf and 27 respondents had both sight and hearing impairments; and almost all respondents reporting internal organ damage also had other impairment/s.

Almost two thirds of respondents lived with partners/spouses or with a partner/spouse and other family members. Nearly a quarter (76/22%) lived alone (compared to 17% of the general UK population aged 50 to 64 [23]; 10% lived with another family member/s (e.g. parents/sibling). Nine respondents lived at home with full-time paid carers or in residential care. The majority of respondents (304/87%) owned their house or flat, compared to 75% of the general UK population aged 50 to 64 (75%) [24].

### Self-reported health problems

The survey asked Thalidomide survivors about additional health problems, over and above their original impairments.

#### Musculoskeletal problems and pain

Almost all (93%) reported musculoskeletal problems (pain and/or loss of movement in joints, neck and/or back). Back problems were the most commonly reported musculoskeletal problem, closely followed by shoulder pain/loss of movement and problems with hands (Table 1). In comparison, less than 20% of adults aged 45–64 in the general population report chronic musculoskeletal conditions [25] and only 15% suffer low back pain [26]. The prevalence of musculoskeletal problems, particularly back problems, is therefore higher amongst UK Thalidomide survivors than in the general population, a pattern also found in Swedish Thalidomide survivors [27]. Many survey respondents reported multiple musculoskeletal problems, with a mean of 4.5 problems and over half reporting five or more problems.

**Table 1:** Prevalence of musculoskeletal problems.

Musculoskeletal Problem	Number/%
Back problems–prolapsed disc; damage to vertebrae; scoliosis and/or muscular pain	254/72%
Shoulder–pain, loss of movement or deterioration of the joint	211/60%
Hands–pain, loss of grip and/or dexterity	210/59%
Arms and wrists–pain, loss of strength and/or movement	197/56%
Neck pain and/or loss of movement	195/55%
Knee–pain or deterioration of the joint	168/48%
Hip–pain, loss of movement or deterioration of the joint	164/46%
Ankles, feet and toes–pain and/or loss of movement	100/28%

Almost half (49%) of respondents reported generalised pain: 26% said this was severe and/or continuous and a further 23% described it as moderate and/or intermittent. Respondents with most severe Thalidomide damage were the most likely to report generalised pain. For some, the cause of the pain was unclear:

*“Chronic neuropathic pain in peroneal nerves—both legs*, *below knee*. *5 years*. *No cause found*. *Does not respond to treatment/pain management medication*.*”* (Survey ID298)

*“I have been suffering with regular bouts of pain in my side*. *I have had several tests done but I am told there is no conclusive reason for the pain*.*”* (Survey ID104)

Two-thirds (66%) of respondents also reported one or more neurological symptom (e.g. tingling and numbness). Two fifths reported severe tiredness/fatigue; this was more common among respondents in Bands 1 and 2, possibly because they were more likely still to be in paid work and therefore less able to rest.

#### Mental health

Half the respondents said that they were currently or had recently experienced depression and/or anxiety (41% reported anxiety and 34% depression). This is in stark contrast to the estimated 20% of adults in the general UK population aged 50–54 reporting common mental health problems (e.g. depression, anxiety or panic) and the estimated 8%-12% adults of all ages reporting depression in any year [28]. The prevalence of poor mental health among UK Thalidomide survivors is also higher than a general population survey of 7500 physically disabled adults (all ages), of whom 19.5% reported depression or mixed depression/anxiety [17]. However, it is very similar to the prevalence of mental health problems among German survivors [11], where face-to-face psychiatric assessments found 47.7% of Thalidomide survivors currently or recently experienced mental health problems.

#### Hearing, sight and dental problems

Deteriorating sight/eye problems were a concern for 43% of respondents and 38% (133) said that they had deteriorating hearing/other ear problems. Although these figures will include normal age-related deterioration, this can cause additional problems for Thalidomide survivors e.g. having very short arms makes putting glasses on and off difficult, whilst wearing glasses can be problematic for those with facial damage (e.g. a missing ear). Thirty-four percent of respondents reported dental health problems. As with Swedish Thalidomide survivors [29], these problems may reflect difficulties with tooth brushing and a history of using teeth to grip and hold.

#### Other health problems

Respondents reported a range of other health problems, with weight management (n = 141/40%), bowel and digestive problems (n = 98/28%), bladder or continence problems (n = 72/20%) and asthma or breathing problems (n = 54/15%) being the most common. Again, these health problems may be more difficult to manage or exacerbated by Thalidomide damage. A recent Japanese study [30] suggests that hypertension is a particular concern because of the difficulties many Thalidomide survivors experience in exercising and managing weight, and the challenge of obtaining accurate blood pressure and body mass index measurements in people with limb impairments.

#### Multimorbidity

**Fig 2:**
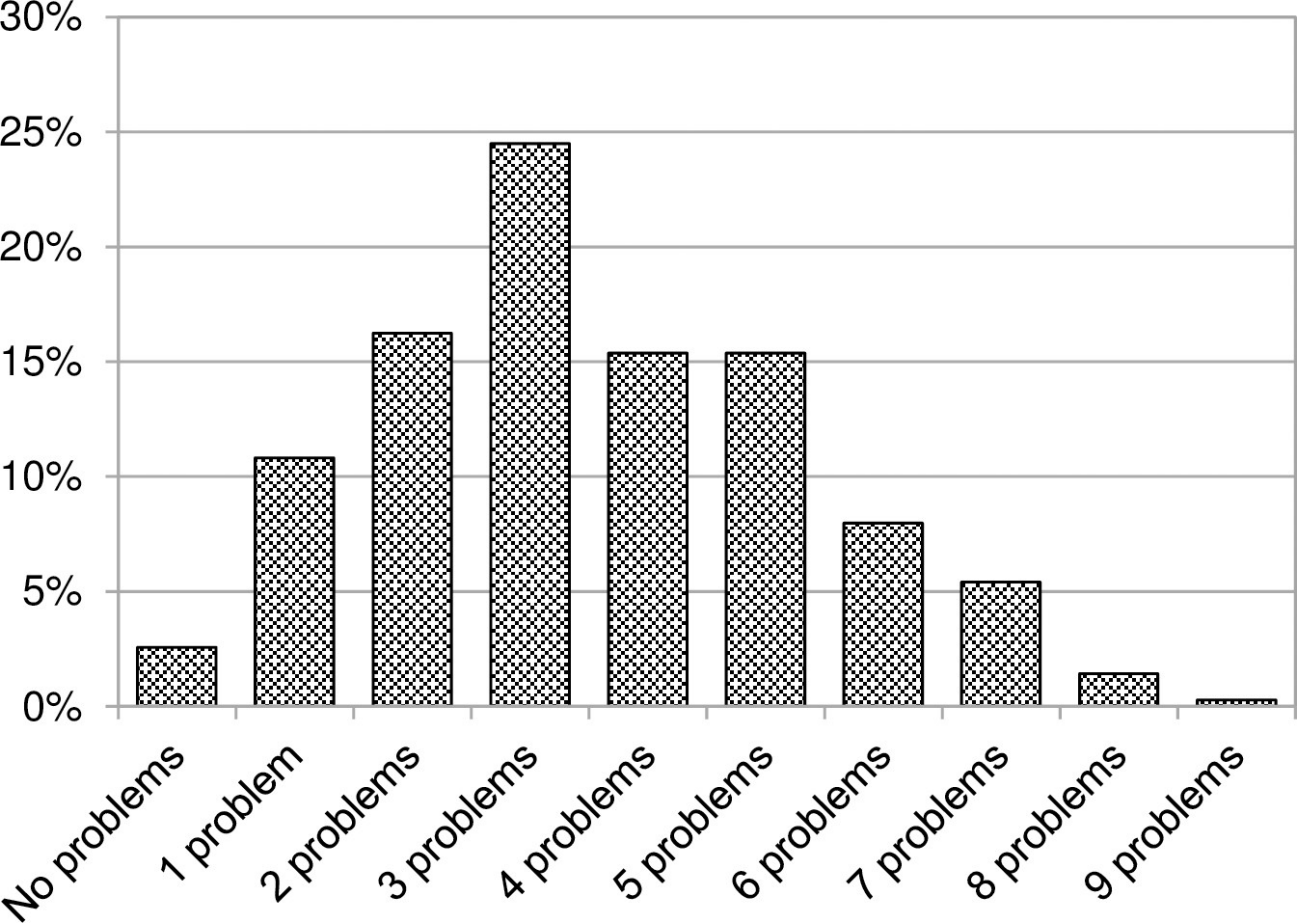
Proportions of Thalidomide survivors reporting multiple health problems.

A high proportion of Thalidomide survivors reported multiple health problems (see Fig 2). Just 3% had no health problem but almost half (46%) reported between four and nine separate problems. Multimorbidity or the presence of multiple diseases in one individual is a growing concern in the UK population as a whole but these results suggest that a higher proportion of Thalidomide survivors are experiencing multimorbidity than would be expected at this point in their life course. For example, in Barnett et al’s [31] study of multimorbidity in Scotland, around a third of patients aged 55 to 59 had two of more morbidities but less than 10% had four or more morbidities. Furthermore, for some Thalidomide survivors the morbidity burden i.e. the overall impact of several conditions on the individual’s *“physiologic reserve”* and functioning [32] is significant. A comment added by one respondent vividly illustrates this:

*“After having operations on both shoulders and left elbow the use of my arms and hand were considerably limited e*.*g*. *dressing*, *washing*, *cooking*, *and most basic household chores*. *Also having a mini stroke has made my balance a bit difficult*. *And then the cancer came along…”* (Survey ID21)

### The impact of health problems on paid work

These widespread health problems were reflected in survey respondents’ employment status. Two-fifths (145/41.3%) were now unable to work because of their disability or health problems (Table 2). Previous research suggests that less than 10% of UK Thalidomide survivors have never worked [33]. As in the general population, there were significant differences between men and women: men were more likely to be working full-time; women were more likely to work part-time; and women were also marginally more likely to be unable to work because of their disability or health problems (69 men compared to 76 women).

**Table 2:** Work situation.

Work Situation–all respondents	Number/%
I’m unable to work because of my disability or health problems	145/41.4%
I work full-time	54/15.4%
I have chosen not to work in order to preserve my health/functioning	45/12.9%
I work part-time because of my disability or health problems	30/8.6%
I work part time in order to preserve my health/functioning	29/8.3%
I work part-time for family or personal reasons	17/4.9%
I’m not working at the moment but would like to	15/4.3%
I’ve chosen not to work for family or personal reasons	8/2.3%
Other (e.g. education)	7/2.0%

The likelihood of not working increased with the severity of impairment: the proportions reporting currently being unable to work increased from 35% of those in Band 1 (least severe) to 75% in Band 5 (most severe); whilst the proportion currently in full-time work decreased from 32% (Band 1) to 8% (Band 5). Moreover, although respondents in impairment bands 1 and 2 were more likely than their peers with more severe impairments to still be in full time work, around a third of them still reported being unable to work because of their disability or health problems. Patterns for those working part-time were less clear.

Comparisons with the general population, and/or with people with disabilities as a whole are difficult, as different sources use different terminology, definitions and age groupings. However, in 2015, 82% of the general population aged 50–54 (78% of women, 86% of men) were in paid work [34] (DWP 2015), suggesting that only 18% of the age group were ‘economically inactive’, compared to 63% of Thalidomide survivors.

**Fig 3:**
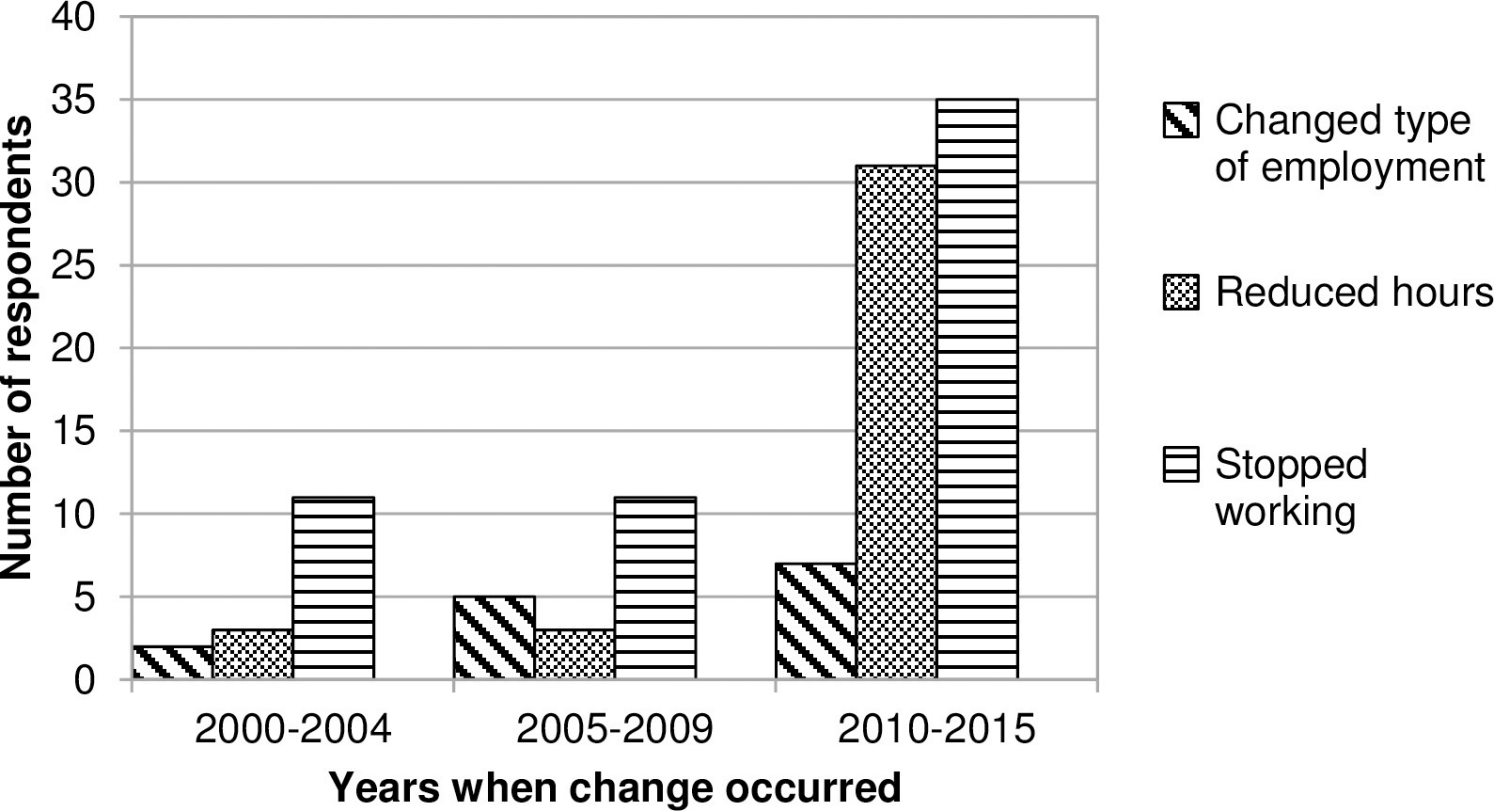
Changes in work situation since 2000. Note: 15 respondents reported more than one change, so appear in more than one category; all had reduced working hours and all but one had changed the type of work they did.

In the UK, disabled people may be four times as likely as their non-disabled peers to be unemployed or involuntarily out of work [35]. However, the lifetime economic activity patterns of Thalidomide survivors appear to be somewhat different from those of other disabled people. First, their current employment patterns do not reflect the lower levels of educational achievement which commonly disadvantage disabled people; their educational achievements were broadly similar to those of the general population of a similar age [36]. The proportion of Thalidomide survivors with no qualifications was similar to those of working age disabled people in general. On the other hand, the proportion with degree-level qualifications was higher than working age disabled people in general [37]. Similar educational qualification patterns have been found among German Thalidomide survivors [11]. Secondly, at each level of educational qualification, there was a range of severity and type of impairment (e.g. the 19 respondents with degrees working full time were spread across all five severity bands and most types of impairment). This suggests that whilst respondents’ ability to work was strongly linked to the severity of their impairment, educational qualifications also had an important influence. Thirdly, the survey showed that Thalidomide survivors’ work situation had changed significantly over the last 10 to 15 years. Overall, 207 respondents (59%) reported changes in their work situation since 2000, with most changes taking place in the last five years (Fig 3). This trend has also been seen amongst Thalidomide survivors in Canada and Germany [9, 38].

*“Increasingly from 2002 onwards*, *at which time I was a Director of a limited company working in excess of 50 hours per week*. *I now struggle to manage 18 hours per week*. *I have now reached the point where stopping work altogether is imminent*.*”* (Survey ID2)

Less severely impaired respondents (Bands 1 to 3) were more likely to have reduced their working hours or changed the type of work they did. Although greater proportions of more severely impaired beneficiaries (Bands 4 and 5) had stopped working since 2000, this change had also occurred across all impairment bands. For example, over three quarters of respondents in bands 1 to 4 were working prior to 2000. By 2015, less than half of all respondents were working and, moving from band 1 to band 5, there was a gradual fall in the proportions still working.

*“2011 I was forced to make a difficult decision to stop working due to continued failing health*.*”* (Survey ID183)

*“In 2009 I had to reduce my hours in line with medical advice*. *In 2010 I had further problems… and in 2011 I was medically retired (not at my request) as my employer had no role for me*.*”* (Survey ID276)

### Thalidomide survivors’ health-related quality of life

**Fig 4:**
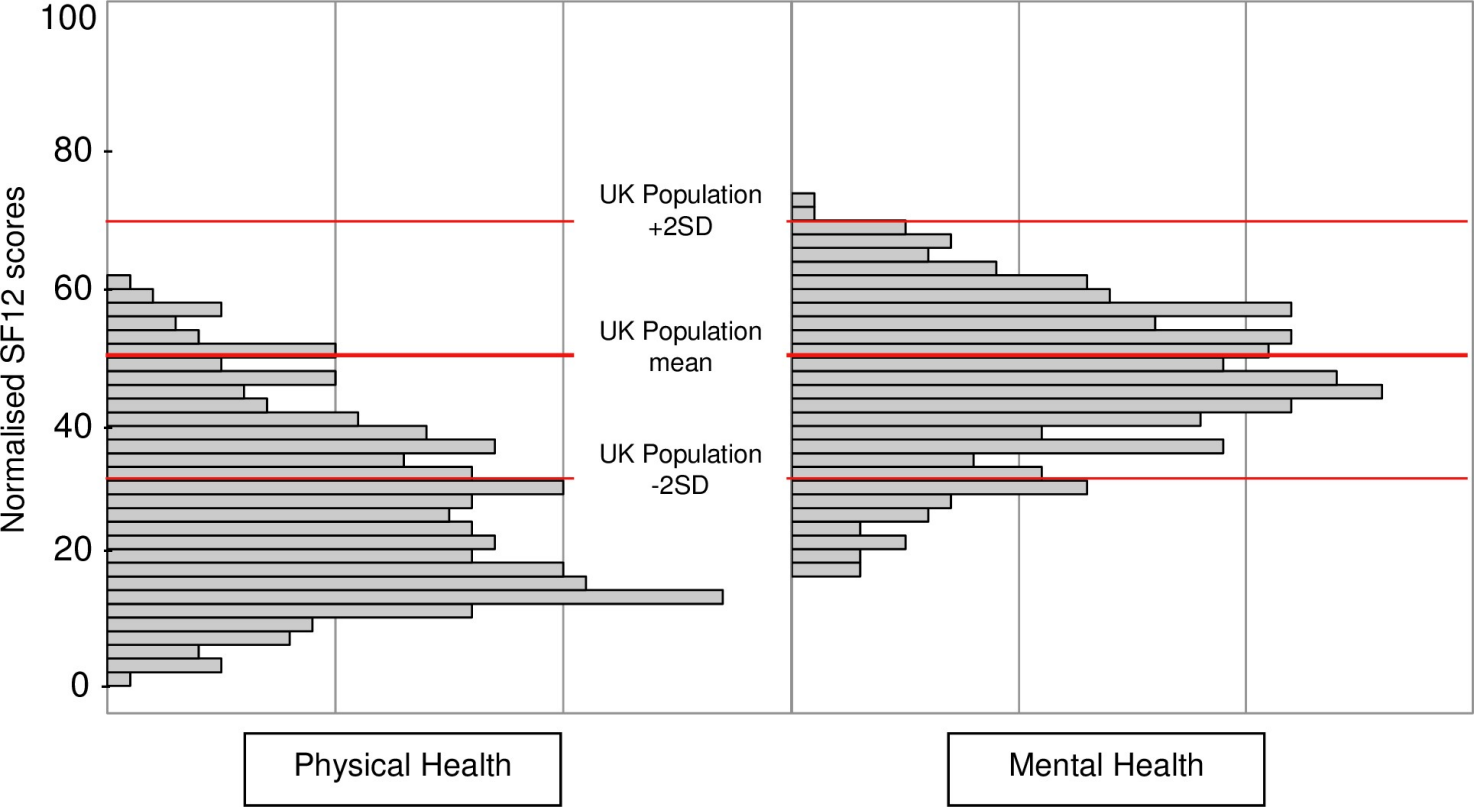
Normalised SF-12 physical and mental health scores for survey group.

The SF12 Health Survey was used to measure health related quality of life. SF12 consists of eight scaled sections (General Health; Pain; Physical Functioning; Role Limitation Physical; Mental Health; Role Limitation Emotional; Social Functioning; Vitality), which can be ‘aggregated’ into two domains: physical health related quality of life and mental health related quality of life. Of the 351 survey respondents, 335 returned SF12 questionnaires that were useable for analysis (i.e. no missing data) and for 285 of these we were able to link SF12 scores to level of impairment (as indicated by the number of impairment points the individual had). Fig 4 shows the range of scores for the survey respondents compared to the general population aged 45–54, based on responses to the Central England Healthy Life Survey [39].

In the physical health domain respondents had a markedly lower average aggregate score than the general population (i.e. a mean of 26.7 compared to 50) indicating that their physical health related quality of life is much poorer. 59.7% (n = 200) of the respondents had a score below 30 i.e. the same as or worse than the 2% of the general population group with the poorest physical health related quality of life, and only 7.5% (n = 25) of the respondents had a score above the average for the general population group.

In the mental health domain the average aggregate score for respondents was 46.5, slightly lower than the general population (score 50). This suggests that on average Thalidomide survivors’ mental health related quality of life is only marginally poorer than their peers in the general population; indeed 40.9% of respondents (n = 137) had a score above the average for the general population. However, 10.7% (n = 36) had a score below 30 i.e. the same as or worse than the 2% of the general population with the poorest mental health related quality of life. Overall men had a higher mean score for physical health (28.6 compared to 24.7) which was significant (t (327) = 2.567, p = 0.01.). Women had a higher mental health score (47.8 compared to 45.1), which was not significant (t (327) = -1.981, p = 0.05).

We also used the SF12 results to examine whether there was any relationship between health-related quality of life and level of impairment. We found that there was a strong negative correlation (*r* = -.276; *p* = .000) between lower SF12 physical health scores and severity of impairment i.e. the *more* severe a respondent’s Thalidomide damage, the poorer their physical health related quality of life was likely to be. In contrast, when we examined the same relationship for mental health related quality of life, we found that the *less* severe a respondent’s Thalidomide damage, the poorer their mental health related quality of life was likely to be i.e. there was a positive (but weaker) correlation between lower SF12 mental health scores and less severe impairment (*r* = .148; *p* = .012).

The responses to the narrative questions in the survey suggested that other factors, in particular being unable to work because of secondary health problems, could be important in explaining variance in both physical and mental health-related quality of life. To examine if this was the case we used hierarchical regression, using three sets of variables: original impairment level (as indicated by the number of impairment points) (step 1); being unable/able to work and qualifications (step 2); and gender and living alone/living with others (step 3). Tables 3 and 4 below show the results for SF12 physical and mental health scores.

**Table 3:** Hierarchical Regression results for SF12 Physical.

	B	*SE* B	B (95% CI)	Sig.
Impairment Level	- 0.194	0.063	-0.31, -0.07	0.002
Unable to Work	11.556	1.572	8.46, 14.65	0.000
Qualifications	- 0.184	0.290	-0.75, 0.38	0.526
Gender	- 3.207	1.471	-6.10, -0.31	0.030
Live Alone	- 0.343	1.755	-3.79, 3.11	0.845

Note: Step 1 Adj *R*^2^ = 0.079; Step 2 Adj *R*^2^ = 0.243, Δ *R*^2^ = 0.169; Step 3 Adj *R*^2^ = 0.251, Δ *R*^2^ = 0.014

**Table 4:** Hierarchical Regression results for SF12 mental health.

	B	*SE* B	B (95% CI)	Sig.
Impairment Level	0.190	0.060	0.07, 0.30	0.002
Unable to Work	5.026	1.497	2.07, 7.97	0.001
Qualifications	- 0.355	0.276	-0.89, 0.18	0.200
Gender	2.077	1.401	-0.68, 4.83	0.140
Live Alone	0.974	1.671	-2.31, 4.26	0.561

Note: Step 1 Adj *R*^2^ = 0.016; Step 2 Adj *R*^2^ = 0.067, Δ *R*^2^ = 0.058; Step 3 Adj *R*^2^ = 0.069, Δ *R*^2^ = 0.009

The model predicting physical health was significant at: step 1—*F* (1, 273) = 24.526, p < .001; step 2 –*F* (3, 271) = 30.337, p < .001; and step 3 –*F* (5, 269) = 19.396 p < .001. The results for SF12 physical show that together the five variables explained 25% of the variance in physical health related quality of life. However, only three variables make a unique statistically significant contribution (i.e. *p* = .05 or less)–a higher level of original impairment, being unable to work, and gender (being male), predicted poorer physical health related quality of life, with being unable to work accounting for most of the variance.

The model predicting mental health was sig at: step 1—*F* (1, 273) = 5.348, p = .021; step 2 –*F* (3, 271) = 7.514, p < .001; and step 3 –*F* (5, 269) = 5.080 p < .001. For mental health related quality of life, the five variables explained just 7% of the variance in SF12 mental health scores, with only two variables–lower level of original impairment and being unable to work, making a unique statistically significant contribution to predicting poorer mental health related quality of life.

## Discussion

Over the past five decades, the lives of UK Thalidomide survivors’ have been documented in the media. They are (rightly) often portrayed as remarkable individuals who have overcome a unique range of physical impairments to lead active and productive lives. However, the findings from this first comprehensive investigation of the health of UK Thalidomide survivors in their early/mid 50s show that in addition to their original impairments, many are now reporting multiple secondary health problems and rapid loss of function, thereby creating the potential for further disabling consequences. The layering of these secondary health problems on to lifelong impairments is causing many Thalidomide survivors to give up paid work prematurely (a trend which has accelerated in the last 10 years), and need more help in their daily lives. From being hitherto relatively independent, they appear to be increasingly out of step with their non-disabled peers at the same stage in the life course.

Musculoskeletal problems were over four times as common amongst Thalidomide survivors as in the general population of a similar age, and a high proportion experience multiple musculoskeletal problems. These problems are leading to loss of function and associated difficulties with activities of daily living and for many this is having a negative impact on their mental wellbeing. This link has been found in other studies of disabled people. Melzer et al. [17] found that the number of Activities of Daily Living/Instrumental Activities of Daily Living that people with disabilities had difficulties with, had an incremental effect on the likelihood of them experiencing depression. The quotation below from a response to the narrative questions in the survey illustrates this effect in relation to Thalidomide survivors:

*“My only normal hand is deteriorating badly*. *I've had 3 operations on it*, *they can't do anything more*. *I'm in pain with it nearly all the time*. *I can't do hardly anything for myself now*. *I'm terrified*. *I'm only 55 –how much worse is it going to get*? *Having one hand I was never disabled but I am now*. *Luckily I have fantastic children who all automatically do everything for me that's needed*. *They cut my food up*, *do up my buttons*, *zips*, *and laces*, *and are amazing but I don't want to be a burden to them*. *Losing your independence is soul destroying*.*”* (Survey ID307)

Alongside secondary physical health problems, half the survey respondents said that they were currently or had recently experienced depression and/or anxiety. This self-reported prevalence of common mental health problems is significantly higher than in the same age group in the general population and higher than adults (all ages), with other physical disabilities. However, it is supported by evidence from clinical studies conducted with Thalidomide survivors in Germany.

The prevalence amongst UK Thalidomide survivors of ‘lifestyle’ diseases and other conditions associated with middle and older age is unclear. However, the practical challenges of preventing and self-managing conditions such as diabetes, can be huge for people with limb difference. A high proportion of survey respondents reported the same risk factors for lifestyle diseases as other people with physical disabilities, notably weight management and difficulties exercising. The risk for Thalidomide survivors may be compounded by the problems of accurately measuring blood pressure and body mass index when people have missing or short limbs [30]. Crucially, many Thalidomide survivors reported multiple health problems. Moreover, a higher proportion of them were experiencing multimorbidity than would be expected at this point in their life course, and for some the morbidity burden appeared significant.

The study also showed that Thalidomide survivors experience significantly poorer physical health related quality of life compared to the general population but their mental health related quality of life was only marginally poorer than their peers in the general population. These findings are consistent with a German study of 186 Thalidomide survivors in North Rhine Westphalia, which used SF36. Peters et al. [11], reported that Thalidomide survivors had a mean aggregate physical score of 29.6 and a mean aggregate mental health score of 47.8. A Swedish study of 31 Thalidomide survivors [40] also found that their physical health related quality of life was significantly lower than the general population, although their mental health related quality of life was similar.

Whilst the more severe a respondent’s original Thalidomide damage, the poorer their physical health related quality of life was likely to be, being unable to work accounted for most of the variance in SF12 scores. This suggests that secondary health problems and associated loss of function may be a far more important influence on physical health related quality of life than original impairment alone. In relation to mental health related quality of life, the narrative responses to the survey suggest that for Thalidomide survivors with lover levels of impairment, who have probably been actively employed for most of their lives, having to give up work (as opposed to choosing not to work) is having a detrimental effect on their mental wellbeing. However, the quantitative analysis showed that being unable to work accounted for just under 7% of the variance in SF12 scores, which implies that psychosocial factors, not explored in the survey, may be of more importance.

Studies of the wellbeing and quality of life of people with disabilities [41, 42], have found that those disabled from birth were likely to have higher subjective wellbeing that those disabled later in life. This study shows that whilst the majority of Thalidomide survivors have lived relatively independent lives, many are now having to adjust to growing disability. For those with mild to moderate impairments in particular, the experience of this change appears to be similar to people disabled later in life. However, Thalidomide survivors (as a group) are in some respects atypical of people with disabilities: they have a similar level of education to their peers in the general population; and at least in recent years, they have been more financially secure due to improved compensation payments and the DH Grant. Moreover, for many any employment disadvantage (as indicated by lower rates of labour market participation) is relatively recent. Nevertheless, at this relatively late stage in life they are experiencing exposure to the environmental and other disadvantages common to many disabled people.

This study provides further evidence of the accumulative impact of disability over peoples’ lifetimes; it demonstrates that whilst from birth conditions like Thalidomide Embryopathy are non-progressive, they are not static; and it highlights the value of a life course perspective in understanding the complex experience of growing older with a disability. Our paper, by describing how impairments change over time, represents a starting point. More theoretically work connecting this to the accumulative potential for disability is required, along with a commitment to using these insights to inform a more nuanced policy, which is able to accommodate the fluid and changing nature of the disabling experience.

## Supporting information

S1 Questionnaire Thalidomide trust health and wellbeing questionnaire 2015. (PDF)

S1 Dataset Health and quality of life of UK Thalidomide survivors. (XLSX)

## References

[1] Berghs M, Atkin K, Graham H, Hatton C, Thomas C. Implications for public health research of models and theories of disability: a scoping review and evidence synthesis, Public Health Research. 2016; 4(8) 27512753

[2] Power A, Bertlett R. Ageing with a learning disability: Care and support in the context of austerity. Social Science and Medicine. 2018; 10.1016/j.socscimed.2018.03.028 29580648

[3] Smithells RW, Newman CGH. Recognition of thalidomide defects. J Med Genet. 1992 29:716–723. 1433232 10.1136/jmg.29.10.716 PMC1016130

[4] Ferber S. Bioethics in Historical Perspective. Palgrave Macmillan; 2013.

[5] Brynner R, Stephens T. Dark Remedy Perseus Publishing; 2001.

[6] World Health Organization. World Report on Disability Geneva: World Health Organization; 2011 Available from: http://www.ncbi.nlm.nih.gov/pubmed/21665020

[7] Scott C. The forgotten victims. Sunday Times, 3 May 2015. Available from: https://www.thetimes.co.uk/article/the-forgotten-victims-s6q3v0q9n88

[8] Newbronner E. and Atkin K. (2018). The changing health of Thalidomide survivors as they age: a scoping review. Disability and Health Journal. 2018; 11: pp184–191. 10.1016/j.dhjo.2017.09.004 29109034

[9] Kruse A, Ding-Greiner C, Beckerr G, Stolla C, Becker A-M, Baiker D. Regular surveys on problems, special needs and care deficiencies of victims of Thalidomide–synopsis of final report presented to the Contergan Foundation for People with Disabilities University of Heidelberg; 2013.

[10] Kayamori R. Post-Thalidomide Syndrome 50 Years On. Japanese Journal of Rehabilitation Medicine. 2013; 50; 957–96.

[11] Peters KM, Albus C, Lungen M, Niecke A Pfaff H, Samel C. Damage to Health, Psychosocial Disorders and Care Requirements of Thalidomide Victims in North Rhine Westphalia from a Long Term Perspective Cologne: Federal Health Centre North Rhine Westphalia; 2015.

[12] Turner RJ, Lloyd DA, Taylor J. Physical Disability and Mental Health: An Epidemiology of Psychiatric and Substance Disorders. Rehabilitation Psychology. 2006; Vol. 51, No. 3, 214–223.

[13] Verbrugge LM, Yang L. Aging with disability and disability with aging. Journal of Disability Policy Studies. 2002; 12(4): 253–67.

[14] Naidoo V, Putnam M, Spindel A. Key focal areas for bridging the fields of aging and disability: findings form the growing older with disability conference. International Journal of Integrated Care. 2012; Oct-Dec, URN:NBN:NL:UI:10-1-113853. 10.5334/ijic.1082 PMC3601515 23593057

[15] Klingbeil H, Baer HR, Wilson PE. Aging with a disability. Arch Phys Med Rehabil. 2004;7:S68–S73. 10.1016/j.apmr.2004.03.014 15221734

[16] Molton IR, Yorkston KM. Growing older with physical disability: a special application of the successful ageing paradigm. J Gerontol B Psychol Sci Soc Sci. 2017; Vol. 72, No. 2: 290–299. 10.1093/geronb/gbw122 27702838

[17] Meltzer H, Bebbington P, Brugha T, McManus S, Rai D, Dennis M, et al Physical ill health, disability, dependence and depression: Results from the 2007 national survey of psychiatric morbidity among adults in England. Disability and Health Journal. 2012; 5: 102–110. 10.1016/j.dhjo.2012.02.001 22429544

[18] Jensen MP, Truitt AR, Schomer K G, Yorkston KM, Baylor C, Molton IR. Frequency and age effects of secondary health conditions in individuals with spinal cord injury: A scoping review. Spinal Cord. 2013; 51: 882–892. 10.1038/sc.2013.112 24126851

[19] LaPlante M. Key Goals and indicators for successful aging of adults with early onset disability. Disability and Health Journal. 2014; 7: S44–S50. 10.1016/j.dhjo.2013.08.005 24456685 PMC3901946

[20] Priestley M. Disability and the Life Course; Global Perspectives Cambridge: Cambridge University Press; 2001.

[21] Jeppsson Grassman E, Whitaker A. Ageing with disability: A lifecourse perspective Bristol: Policy Press at the University of Bristol; 2013.

[22] Elder GH Jr., Johnson MK, and Crosnoe R. The emergence and development of life course theory In Mortimer JT, and Shanahan MJ, editors. Handbook of the life course. New York: Springer; 2004 pp. 3–19.

[23] Office of National Statistics (2014). Living Alone in England and Wales. See: http://www.ons.gov.uk/ons/rel/census/2011-census-analysis/do-the-demographic-and-socio-economic-characteristics-of-those-living-alone-in-england-and-wales-differ-from-the-general-population-/sty-living-alone-in-the-uk.html

[24] Office of National Statistics (2013). Home ownership and renting in England and Wales–detailed characteristics. See: http://www.ons.gov.uk/ons/rel/census/2011-census/detailed-characteristics-on-housing-for-local-authorities-in-england-and-wales/short-story-on-detailed-characteristics.html

[25] Office for National Statistics. General Household Survey (2007). Available from: http://www.statistics.gov.uk/downloads/theme_compendia/GHS07GeneralHouseholdSurvey2007.pdf

[26] Hoy D, March L, Brooks P, Blyth F, Woolf A, Bain C, et al The global burden of low back pain: estimates from the Global Burden of Disease 2010 study. Annals of the Rheumatic Diseases. Published online 3 24 2014. 10.1136/annrheumdis-2013-204428 24665116

[27] Ghassemi Jahani SA, Danielsson A, Karlsson J, Brisby H. Degenerative Changes in the Cervical Spine Are More Common in Middle-Aged Individuals with Thalidomide Embryopathy than in Healthy Controls. PLoS ONE. 2016; 11(5): e0155493 10.1371/journal.pone.0155493 27175919 PMC4866686

[28] Mental Health Foundation. The Fundamental Facts London: Mental Health Foundation; 2007.

[29] Ekfeldt A, Carlsson GE. Dental status and oral function in an adult group of subjects with thalidomide embryopathy—A clinical and questionnaire study. Acta Odontologica Scandinavica. 2008; 66(5), 300–306. 10.1080/00016350802307638 18720052

[30] Shiga T, Shimbo T, Yoshizwa A. Multicentre Investigation of Lifestyle-Related Diseases and Visceral Disorders in Thalidomide Embryopathy at around 50 years of age. Birth Defects Research (Part A). 2015; 103:787–793. 26033770 10.1002/bdra.23363 PMC5157726

[31] Barnett K, Mercer SW, Norbury M, Watt G, Wyke S, Guthrie B. Epidemiology of multimorbidity and implications for health care, research, and medical education: a cross-sectional study. Lancet. 2012; 380: 37–43 10.1016/S0140-6736(12)60240-2 22579043

[32] Valderas JM, Starfield B, Sibbald B, Salisbury C, Roland M. Defining Comorbidity: Implications for Understanding Health and Health Services. Annals of Family Medicine. 2009; 7(4), 357–363. 10.1370/afm.983 19597174 PMC2713155

[33] Newbronner E, Baxter M. Health, Quality of Life and Employment amongst Thalidomide-affected People–Evidence from the UK. Firefly Research/The Thalidomide Trust; 2015.

[34] Department of Work and Pensions (2015). Employment statistics for workers aged 50 and over, by 5-year age bands and gender: from 1984 to 2015 DWP; London.

[35] Papworth Trust. Disability in the United Kingdom 2014: Facts and Figures. Cambridge; 2014.

[36] Office for National Statistics (2011). Highest levels of qualification across England and Wales. Available from: http://www.ons.gov.uk/ons/rel/census/2011-census-analysis/local-area-analysis-of-qualifications-across-england-and-wales/info-highest-qualifications.html

[37] Office for National Statistics Office for National Statistics (2012). Labour Force Survey. Available from: http://webarchive.nationalarchives.gov.uk/20160107075206/http://www.ons.gov.uk/ons/rel/lms/labour-market-statistics/november-2012/statistical-bulletin.html

[38] Vermette N, Benegabi M. Study on the current living conditions of Canadian Thalidomide survivors and projections for the future Thalidomide Victims Association of Canada; 2013.

[39] Jenkinson C, Wright L, Coulter A. Quality of Life measurement in health care A review of measures, and population norms for the UKSF-36, Oxford: Health Services Research Unit; 1993.

[40] Ghassemi Jahani SA, Karlsson J, Brisby H, Danielsson AJ. Health-related quality of life and function in middle-aged individuals with thalidomide embryopathy. J Child Orthop. 2016; 10:691–703. 10.1007/s11832-016-0797-6 27854003 PMC5145847

[41] Uppal S. Impact of the timing, type and severity of disability on the subjective wellbeing of individuals with disabilities. Social Science and Medicine. 2006; 63: 525–539. 10.1016/j.socscimed.2006.01.016 16530905

[42] Kemp BJ, Mosqueda L. Ageing with a Disability Baltimore: The Johns Hopkins University Press; 2004.

