# Supplementary material for: The health and quality of life of Thalidomide survivors as they age – Evidence from a UK survey
Source: PLoS One. 2019 Jan 16;14(1):e0210222. doi: 10.1371/journal.pone.0210222 (PMC6334953; doi:10.1371/journal.pone.0210222)
Supplement: S1 Questionnaire — (PDF) [file pone.0210222.s001.pdf]

Firefly
Illuminating Research

The Thalidomide Trust

Health and Wellbeing Survey 2015

About You

At the end of the survey we will ask you for your name. It would be extremely helpful to us if you could give your name but if you would prefer to complete the survey anonymously, that's fine.

Are you?	Male	Female

What is the highest level of education qualification you have obtained (please tick one box)?			
Degree or higher degree (e.g. MA, PhD)		ONC/BTEC or NVQ Level 3	
Diploma or professional qualification (e.g. Registered Nurse)		O Level or GCSE equivalent (Grade A-C)	
A Levels or Highers		O Level or GCSE equivalent (Grade D-G)	
HNC/HND or NVQ Level 4		No formal qualifications	
Other (please describe):			

Family and Housing

Q1 Which of the following best describes your home circumstances (please tick)?

I live alone	
I live with my partner/spouse	
I live with my partner/spouse and other family members (e.g. children)	
I live with another family member (e.g. parent or sibling)	
Other (please describe):	

Q2 Which of the following best describes your housing situation (please tick)?

I live in a house/flat which I (or my partner/family) own	
I live in a private rented house/flat	
I live in a housing association or local authority house/flat	
I live in a residential care home	
Other (please describe):	

Q3 How many years have you lived in your current home?

Q4 Are you planning any adaptations to your home ***in the next year***?

Question is not applicable to me	
No, I have done all the adaptations required to meet my current needs	
No, I can’t afford to do any adaptations in the next year	
Yes, major adaptations e.g. new kitchen, an extension, other structural changes	
Yes, minor adaptations e.g. new door handles, repositioning electrical sockets	
Not sure/don't know	
Is there anything else you would like to tell us about adaptations to your home?	

Q5 If you think you will need to move home ***in the next 5 years*** what are the main reasons for this (please select all that apply)?

Question is not applicable to me - I don't think I will need to move home	
I need a home which is fully adapted for my disabilities/health problems	
I need a smaller house/garden	
I need a home with no stairs e.g. bungalow or flat	
I need a larger house	
I need a house with accommodation for a live-in personal assistant	
I need to release some capital from the value of my home	
I need to move nearer to a family member	
I need to move nearer to shops and services	
Other (please describe):	

Q6 Which, if any, of the following difficulties might affect your ability to move home (please tick all that apply)?

Question is not applicable to me - I don't think I will need to move home	
The cost of buying *and* adapting a new home	
Getting a mortgage/increasing your existing mortgage	
Finding the right property	
Selling your existing home	
Finding a suitable property to rent	
My local authority/housing association accepting that my needs are not being met in my current home	
Not sure/don’t know	
Other (please describe):	

Work and Pensions

Q7 Which of the following best describes your work situation (please tick one)?

I work full-time	
I work part-time because of my disability or health problems	
I work part time in order to preserve my health/functioning	
I work part-time for family or personal reasons	
I’m not working at the moment but would like to	
I’m unable to work because of my disability or health problems	
I have chosen not to work in order to preserve my health/functioning	
I’ve chosen not to work for family or personal reasons	
I’m in full or part-time education	
Other (please describe):	

Q8 ***Since the year 2000***, has your work situation changed in any of the following ways because of your Thalidomide-related disability and/or health problems?

My working situation has not changed	
I have reduced my working hours	
I have changed the type of work I do	
I have stopped working	

If your working situation has changed please tell us in what year(s) this occurred.

Q9 If you currently work full or part time, ***in the next 5 years*** do you think your Thalidomide-related disabilities and/or health problems will require you to change your work situation in any of the following ways?

Question does not apply to me as I am not currently working	
Reduce my working hours?	
Change the type of work I do?	
Stop working?	
I don't expect my work situation to change because of my Thalidomide-related disabilities and/or health problems	

Q10 If you have paid, or are currently paying into an employer/company or private pension (i.e. additional to the normal state pension) approximately how many years of contributions do you have?

Q11 Is there anything else you would like to tell us about your work or pension situation?

Original Thalidomide impairments

We would like to understand how the health and wellbeing of beneficiaries with different types of impairments is changing. The categories in the table below are just intended to provide us with a simple picture and are not comprehensive.

Q12 Original Thalidomide impairments - damage to limbs (please tick all the categories below that you feel apply to you):

	**Left side of body**	**Right side of body**
Minimal or no arm		
Arm shorter than elbow length		
Arm longer than elbow length		
Arm normal length		
Misshapen hand/missing digits		
Misshapen shoulder joint		
Minimal or no leg		
Leg shorter than knee length		
Leg longer than knee length		

Leg normal length		
Misshapen foot		
Misshapen hip		
Misshapen knee		

Q13 Original Thalidomide impairments - other damage (please tick all the categories below that you feel apply to you):

Scoliosis (curved spine)	
Damage to face and/or outer ear (including facial palsy)	
Totally deaf	
Partially deaf	
Totally blind	
Partially sighted	
Damage to heart	
Missing or damaged kidneys	
Damage to digestive system/bowels	
Damage to reproductive organs	
Damage to nervous system	
No 'other damage'	
Other impairments/damage (please describe if you wish):	

Mobility and Equipment

Q14 Do you use any of the following to help you with your mobility? (please tick)

	All of the time	Some of the time	None of the time
Manual wheelchair			
Electric wheelchair			
Mobility scooter			
Prosthetic limbs			

Q15 Do you use a hearing aid/s and/or have hearing implants?

Yes	No

Q16 Do you have a car or van? (please tick all that apply)

I don't have a car or van	
I have a car/van with no adaptations	
I have a car/van with minor adaptations	
I have a car/van with major adaptations	

Q17 Is there anything else you would like to tell us about your mobility or the equipment/vehicles you use e.g. quality of wheelchairs, cost of adapting a car?

Health Problems

Q18 Are you currently experiencing, or have you recently had, any of the following health problems (please tick all that apply to you):

Back problems - prolapsed disc; damage to vertebrae; scoliosis		Anxiety	
Back problems – muscular pain and/or spasms		Alcohol or drug misuse	
Neck pain and/or loss of movement		Other mental health problems	
Shoulder - pain, loss of movement or deterioration of the joint		Generally poor emotional health	
Arms and wrists - pain, loss of strength and/or movement		Generalised pain – severe and/or continuous	
Hands - pain, loss of grip and/or dexterity		Generalised pain – moderate and/or intermittent	
Hip - pain, loss of movement or deterioration of the joint		Severe tiredness/fatigue	
Knee - pain or deterioration of the joint		Tingling/pins and needles	
Ankles, feet and toes - pain and/or loss of movement		Numbness/Loss of feeling	
Problems with the fit or use of prosthetic limbs		Sensations of extreme heat or cold	
Deteriorating sight/eye problems		Heart problems	
Deteriorating hearing/other ear problems		Kidney problems	
Problems with balance/falls		Bladder or continence problems	
Dental health problems		Asthma or breathing problems	
Weight management problems		Diabetes	
Bowel or digestive problems		Stroke/TIA	
Cancer		I have no health problems	
Depression			

Are there any other health problems you would like to tell us about?

Q19 If your health or wellbeing has improved recently what has made the difference e.g. weight loss, successful treatment.

Use of Health Services

Q20 Which, if any of the following health treatments have you had in the ***past 10 years***:

Shoulder replacement/surgery		Physiotherapy - Private	
Wrist/arm surgery		Acupuncture	
Hip replacement/hip surgery		Osteopathy	
Knee replacement/surgery		Chiropractic	
Bariatric surgery for weight problems		Therapeutic Massage	
Treatment to relieve pain e.g. injections		Treatment for depression and/or anxiety, including counselling	
Prescription pain medication		Counselling for other emotional issues	
Back surgery		Treatment for alcohol or drug dependence	
Physiotherapy - NHS		No health treatments	
Are there any other health treatments you would like to tell us about?			

Q21 How well does your GP/GP surgery understand your Thalidomide damage and how it affects you?

Fully understands	Partly understands	Doesn't understand	Not sure/don't know

Q22 If you had a Thalidomide-related health problem do you think your GP would be willing to seek advice from a specialist with knowledge of Thalidomide damage and/or from the Thalidomide Trust?

Yes	No	Not sure/don't know

Q23 ***In the past 5 years*** have you experienced any of the following problems with healthcare services (please select all that apply):

Lack of knowledge/understanding of Thalidomide damage in general amongst health professionals	
Lack of knowledge/understanding of my impairments amongst health professionals	
Lack of involvement/choice in decisions made about my care and treatment	
Difficulties/delays in seeing a suitably experienced health professional/specialist	
Misdiagnosis or delayed diagnosis	
Delays in getting treatment	
Incorrect or inappropriate treatment	
Inflexible care or treatment (e.g. blocks of physiotherapy treatments rather than on-going care)	
Problems with the quality or choice of equipment (e.g. wheelchairs, hearing aids, prosthetics) available through the NHS	
I have not experienced any problems with healthcare services	
Other (please describe)	

Social Care Support

Q24 Do you get local authority funded social care (i.e. personal budget, direct payment or home care services)? Yes | No

If you answered 'Yes' to Q24 please continue with Q25, if 'No' go to Q30:

Q25 Do you feel the level of local authority funded support you get is enough to meet your needs? Yes | No

Q26 Has your local authority funded care package or personal budget/direct payment been reduced in the last 5 years? Yes | No

Q27 Do you pay for additional time/services from your own income? Yes | No

Q28 Do you think you will need more support in the next 5 years? Yes | No

Q29 Is there anything you would like to tell us about the quality and/or availability of the social care services you get?

If you answered 'No' to Q24 continue with Q30, if 'Yes' go to Q32:

Q30 Do you buy support (e.g. personal assistance, help in the home, gardening/DIY) privately? Yes | No

Q31 Do you think you will need to apply for local authority funded social care in the next 5 years? Yes | No

Mental Well-being

We know that many beneficiaries feel they have to *'put a brave face on'*. So, the next few questions are designed to help us get a better picture of how people are really feeling/coping emotionally.

Q32 For each of the following statements ***please circle*** the number that best reflects how you feel now:

Statements	None of the time	Rarely	Some of the time	Often	All of the time
I've been feeling optimistic about the future	1	2	3	4	5
I've been feeling useful	1	2	3	4	5
I've been feeling relaxed	1	2	3	4	5
I've been dealing with problems well	1	2	3	4	5
I've been thinking clearly	1	2	3	4	5
I've been feeling close to other people	1	2	3	4	5
I've been able to make up my own mind about things	1	2	3	4	5
I've been able to be honest about how I am feeling/coping	1	2	3	4	5

Q33 Thinking about your emotional well-being, ***compared to five years ago*** how well do you feel you are coping now with the demands of everyday life (please tick)?

Much worse than 5 years ago	
Worse than 5 years ago	
About the same as 5 years ago	
Better than 5 years ago	
Much better than 5 years ago	

Q34 Thinking about your social life, including social activities and relationships with family, friends and neighbours, which of the following statements best describes your current situation (please tick):

My social life is as good as it could be and I never feel lonely or isolated	
My social life is good but I occasionally feel lonely or isolated	
My social life is ok but I sometimes feel lonely or isolated	
My social life is poor and I often feel lonely or isolated	
My social life is very poor and I feel lonely or isolated most of the time	

Health Related Quality of Life Questions

For each of the following questions, ***please circle*** the word or phrase (in the unshaded boxes) that best describes how your health is now or how you are feeling now. Please try to complete all the questions in this section even if some of the wording does not completely fit your situation, because they are standard questions used internationally to measure health related quality of life.

Q35 In general, would you say your health is:				
Excellent	Very good	Good	Fair	Poor

The following two questions are about activities you might do during a typical day. Does ***your health now limit*** you in these activities? If so, how much?		
Q36 ***Moderate activities***, such as moving a table, pushing a vacuum cleaner or light gardening		
Yes, limited a lot	Yes, limited little	No, not limited at all
Q37 ***More strenuous activity*** such as briskly propelling your wheelchair, climbing several flights of stairs or mowing the lawn.		
Yes, limited a lot	Yes, limited little	No, not limited at all

During the ***past 4 weeks***, how much of the time have you had any of the following problems with your work or other regular daily activities ***as a result of your physical health***?				
Q38 ***Accomplished less*** than you would like				
All of the time	Most of the time	Some of the time	A little of the time	None of the time
Q39 Were limited in the ***kind*** of work or other activities you are able to do				
All of the time	Most of the time	Some of the time	A little of the time	None of the time
During the ***past 4 weeks***, how much of the time have you had any of the following problems with your work or other regular daily activities ***as a result of any emotional problems*** (such as feeling depressed or anxious)?				
Q40 ***Accomplished less*** than you would like				
All of the time	Most of the time	Some of the time	A little of the time	None of the time
Q41 Did work or activities ***less carefully than usual***				
All of the time	Most of the time	Some of the time	A little of the time	None of the time
Q42 During the ***past 4 weeks***, how much did ***pain*** interfere with your normal work (including both work outside the home and housework)?				
Not at all	A little bit	Moderately	Quite a bit	Extremely

These three questions are about how you feel and how things have been with you ***during the past 4 weeks***. For each question, please give the one answer that comes closest to the way you have been feeling. How much of the time during the ***past 4 weeks:***

Q43 Have you felt calm and peaceful?				
All of the time	Most of the time	Some of the time	A little of the time	None of the time

Q44 Did you have a lot of energy?				
All of the time	Most of the time	Some of the time	A little of the time	None of the time

Q45 Have you felt downhearted and depressed?				
All of the time	Most of the time	Some of the time	A little of the time	None of the time

Q46 During the ***past 4 weeks***, how much of the time has your ***physical health or emotional problems*** interfered with your social activities (like meeting friends, visiting relatives etc.)?				
All of the time	Most of the time	Some of the time	A little of the time	None of the time

Q47 When you think about your future which of the following areas of your life are of ***most concern*** to you?

	Most concern	Some concern	No concern
Housing and adaptations			
Long term living arrangements (e.g. residential care)			
Personal assistance and help in the home			
Family and personal relationships			
Social activities/social life			
Your working situation			
Pension provision			
Mobility			
Your physical health			
Your emotional health			
Other (please describe):			

Q51 Is there anything else you would like to tell us about your health and well-being or any issues/concerns you would like to highlight?

Q52 Would you be willing to answer a few more questions about the topics covered in this questionnaire, either on the telephone or in an informal discussion? Yes | No

It would be enormously helpful if you could give your name as it will make it easier for us to explore how the needs and experiences of beneficiaries vary and enable Firefly Research to get in touch with you to follow up this survey (if you said 'Yes' to Q52). It also means that we won't bother you with unnecessary reminders.

If you do give your name your answers will still be ***completely confidential*** *and will only be seen by Firefly Research.*

Name:	

Thank you again for helping us with this survey.
